# Physical activity and eating behaviors patterns associated with high blood pressure among Chinese children and adolescents

**DOI:** 10.1186/s12889-023-16331-1

**Published:** 2023-08-09

**Authors:** Jiaoyan Chen, Yuanyuan Wang, Wenxiu Li, Ya Zhang, Ruiyao Cao, Xingwang Peng, Juan Zhang, Keke Liu, Mei Han, Lianguo Fu

**Affiliations:** Department of child and adolescent health, School of public health, Bengbu Medical College, 2600 East Sea Avenue, Bengbu Anhui, Bengbu, Anhui, 233030 PR China

**Keywords:** Physical activities pattern, Eating behaviors pattern, Pediatric hypertension, Children, Adolescents

## Abstract

**Background:**

Physical activity and eating behavior are associated with hypertension in children and adolescents. Revealing the associations between physical activity patterns, eating behavior patterns and high blood pressure (HBP) could help improve the problem of hypertension from the actual children’s physical activities and eating behaviors.

**Methods:**

A total of 687 students aged 8–15 years were selected from two nine-year primary and secondary schools using stratified cluster random sampling method. The students’ body height, weight, systolic blood pressure (SBP) and diastolic blood pressure (DBP) were measured, and their physical activity time and eating behaviors were surveyed by using CLASS questionnaire and self-made eating behavior questionnaire, respectively. Exploratory factor analysis (EFA) was used to extract moderate to vigorous physical activity factor (MVPAF), sedentary activity factor (SAF), healthy eating behavior factor (HEBF), unhealthy eating behavior factor (UHEBF). MVPAF ≥ SAF was defined as moderate to vigorous physical activity pattern (MVPAP), MVPAF < SAF was defined as sedentary activity pattern (SAP). HEBF ≥ UHEBF was defined as healthy eating behavior pattern (HEBP), while the opposite was defined as unhealthy eating behavior pattern (UHEBP). Lifestyles includes physical activity patterns and eating behavior patterns.

**Results:**

The overall prevalence of hypertension was 5.8% (40/687), and was 5.69% (21/369) in boys and 5.97% (19/318) in girls, respectively. The MVPAF and UHEBF in boys were significantly higher than those in girls (*P* < 0.01), while the SAF in girls was significantly higher than that in boys (*P* < 0.05). The SAF was positively correlated with SBP in girls (*β*(*SE*) = 0.14 (0.50), P = 0.016), and was positively correlated with SBP (*β*(*SE*) = 0.21 (1.22), P = 0.000 and DBP (*β*(*SE*) = 0.14 (0.49), P = 0.006) in boys. The MVPAF was negatively correlated with DBP (*β*(*SE*)=-0.11 (0.40), P = 0.022) in boys. In boys, the SAP increased the risks of HBP (OR (95% CI):3.34 (1.30–8.63)) and high DBP (OR (95% CI):3.08 (1.02–9.34)) compared with MVPAP.

**Conclusion:**

Compared with the boys with MVPAP, boys with SAP may increase the risks of HBP and high DBP. The SAF may be positively associated with SBP in boys and girls, while the MVPAF may be negatively associated with DBP in boys.

## Introduction

Pediatric hypertension is prevalent worldwide and has become an important public health problem. The survey in 2015 showed that the global prevalence of hypertension in children aged 6 years and 14 years was 4.32% and 7.89%, respectively [1]. Childhood HBP can continue to progress to adulthood hypertension, and increase the risk of subclinical cardiovascular disease (target organ damage) in adulthood [2, 3]. Studies have found that physical activities and eating behaviors are associated with hypertension in children and adolescents [4–6].

Regular moderate to vigorous physical activity is considered to be beneficial to control blood pressure. Studies showed that the planned physical activity lowered SBP and DBP in children [7–9]. However, there are also lots of evidences that sedentary activity behaviors, such as writing, looking at electronic screens, put children at risk for elevated blood pressure[10–12]. Carvalho et al. showed that playing video games and watching television increased levels of SBP and DBP [13], and the combination with obesity increased the risk of hypertension in children [14]. In fact, children’s daily physical activity includes both moderate to vigorous and sedentary activities, and their daily physical activity habits may be dominated by moderate to vigorous pattern or sedentary activity pattern (such as a student who mainly has moderate to vigorous physical activities, but also has sedentary activities). Which may provide a better insight into the relationship between physical activity and blood pressure from the actual children’s physical activities.

Eating fruits, vegetables and foods with low fat, sugar and sodium plays an important role in the treatment of hypertension and prevention of associated target organ damage in children and adolescents [15, 16]. Genovesi et al. [17] reported that excessive intake of sugar and salt was an important role in the pathogenesis of hypertension in children. Studies have shown that diets with high total fat and saturated fat will prevent nitric oxide production in the vascular endothelium, which is not conducive to vascular expansion, oxidative stress and repair of inflammation [18, 19]. In fact, children may have both healthy and unhealthy eating behaviors every day, which may be manifested as a pattern dominated by healthy or unhealthy eating behaviors (such as a student who mainly has healthy eating behavior but also has unhealthy eating behavior). However, the associations of eating behavior patterns with HBP in children have also been rarely reported.

The previous studies have only reported the relationship between certain physical activities (such as running, playing basketball, etc.) or each type of physical activities (such as moderate to vigorous physical activities, or sedentary activities) and blood pressure, and the relationship between certain dietary behaviors (such as eating breakfast, drinking carbonated drinks, etc.) or certain dietary behaviors (such as healthy eating behaviors or unhealthy eating behaviors) and blood pressure. However, children’s lifestyle should be comprehensive pattern of physical activity or eating behaviors. There are few reports on the relationship between physical activity patterns or eating behavior pattern and blood pressure. Therefore, the purpose of this study was to reveal the associations between physical activity patterns, eating behavior patterns and HBP in Chinese children and adolescents.

## Participants and methods

### Participants

The sample size was determined according to the cross-sectional design of the current situation. $n = \mu \frac{{{\alpha ^2}}}{2}p(1 - p)/{\delta ^2}$ (where α = 0.05, δ = 0.03, and p = 0.184 is the prevalence of hypertension[20]) The sample size should be 641 (641 = 1.96 × 1.96 × 0.184 × (1–0.184)/0.03/0.03), however, the 5% of sample size needed to be increased for sampling error. Therefore, the minimum sample size was 673 (673 = 641 × (1 + 1.05)). A total of 687 students aged 8–15 years were selected from two nine-year primary and secondary schools using stratified cluster group random sampling method (stratified by grade, grouped by class). The study was approved by the Medical Ethics Committee of Bengbu Medical College [(2015) 003] and conducted in accordance with the Declaration of Helsinki. Participants cooperated voluntarily as well as their guardians signed an informed consent form.

### Body measurements

Children’s body height, weight, SBP, and DBP were measured by medical staff who have received standardized training. When participants were measured, they had an empty stomach, and dressed in light clothes and barefoot. Height were measured using Mechanical height measure, and the readings were accurate to 0.1 cm. An electronic scale was used to measure weight, and the reading was accurate to 0.1 kg. The children with overweight or obesity were screened according to body mass index reference norm for screening overweight and obesity in Chinese children and adolescents[21]. Before the measuring participants’ blood pressure, they rested quietly for more than 10 min. The right-arm blood pressure in sitting position was measured twice repeatedly with an interval of 2 min using mercury sphygmomanometer. Korotkoff I sound was defined as the SBP and Korotkoff V sound was defined as the DBP. If the difference in blood pressure values between the two measurements was > 4 mm Hg, a third measurement was taken, and the average of the two closest blood pressure values was used as the final blood pressure value. Using HBP screening criteria of “WS/T 610–2018 7–18 years old children and adolescents high blood pressure screening threshold” standard: SBP or DBP ≥ P_95_ blood pressure value in the crowd with same sex-age-height grade[22].

#### Physical activity time survey

The CLASS questionnaire [23] was used to survey children’s moderate to vigorous and sedentary activities time (minutes) in the recent week, including playing football, basketball, tennis, table tennis, badminton, volleyball, and running, roller skating, jumping rope, swimming, cycling, dancing, doing gymnastics, doing housework, playing with pets, playing with skateboards or scooters, walking, walking with pets, doing group games, and other moderate to vigorous physical activities, doing homework, attending tutorial classes, watching TV/DVDs/movies, playing video/computer/mobile games, surfing the internet, playing toys indoors, sitting and chatting, playing musical instruments, playing chess/card games, reading books, drawing, and taking transportation to and from school. EFA was used to extract MVPAF and SAF. MVPAF ≥ SAF was defined as MVPAP, otherwise it was defined as SAP. Factor loadings of MVPAF and SAF were showed in Table 1.

**Table 1:** Factor loadings for physical activity time, eating behaviors using Varimax with Kaiser Normalization

Physical activities (minutes/week)			Eating behaviors (times/week)		
Variables	MVPAF	SAF	Variables	UHEBF	HEBF
Doing housework	0.560	0.061	Western-style fast food	0.704	-0.187
Cycling	0.488	0.026	Carbonated drinks	0.681	-0.104
Running	0.475	0.210	High-calorie snacks	0.666	-0.020
Roller skating	0.461	0.116	Fried food	0.650	-0.019
Walking	0.452	0.318	Eating out	0.556	-0.120
Playing with skateboards or scooters	0.432	0.016	Pickled pickles	0.483	0.000
Playing group games	0.426	0.130	Late night snack	0.468	0.055
Playing football	0.424	-0.087	Fruits	-0.053	0.756
Playing badminton	0.405	0.003	Milk	0.001	0.724
Playing table tennis	0.384	-0.032	Fresh Vegetables	-0.149	0.716
Playing toys indoors	0.376	0.138	Eggs	0.071	0.643
Playing chess and cards	0.360	0.049	Breakfast	-0.126	0.530
Other moderate - high intensity physical activity	0.307	0.039			
Swimming	0.294	0.164			
Jumping rope	0.294	0.036			
Walking with pets	0.273	0.244			
Playing basketball	0.250	0.026			
Playing with pets	0.233	0.189			
Doing gymnastics	0.232	-0.076			
Art calligraphy painting pottery	0.230	0.113			
Playing volleyball	0.223	-0.055			
Playing tennis	0.097	-0.078			
Surfing the Internet	-0.155	0.723			
Watching TV/DVDs/movies	-0.028	0.649			
Playing video/computer / mobile games	-0.069	0.634			
Doing homework	-0.072	0.479			
Sitting and chatting	0.211	0.417			
Dancing	0.024	0.395			
Attending tutorial classes	0.026	0.393			
Reading books	0.214	0.386			
Playing a musical instrument	0.137	0.287			
Doing transportation to and from school	0.069	0.265			

MVPAF, moderate to vigorous physical activity factor; SAF, sedentary activity factor; UHEBF, unhealthy eating behavior factor; HEBF, healthy eating behavior factor

#### Eating behaviors survey

Self-made eating behaviors questionnaire was used to survey the frequency of 12 types of eating behaviors, including breakfast, fruits, fresh vegetables, dairy, eggs, eating out, fried foods, western-style fast food, high-calorie snacks, carbonated drinks, late-night snacks, and pickled pickles. Each eating behavior assigned 6 grades including never (0 points), 1 time per month (0.25 points), 2 time per month (0.5 points), 1–3 times per week (2 points), 4–6 times per week (5 points), and 1 time per day (7 points)[20]. EFA was used to extract HEBF and UHEBF. HEBF ≥ UHEBF was defined as HEBP, while the opposite was defined as UHEBP. Factor loadings of HEBF and UHEBF were showed in Table 1.

#### Lifestyles

Lifestyles include physical activity patterns and eating behavior patterns, and we classify lifestyles into the following four types:

Lifestyle 0 (MVPAP + HEBP): A lifestyle that includes moderate to vigorous physical activity pattern and healthy eating behavior pattern.

Lifestyle 1 (MVPAP + UHEBP): A lifestyle that includes moderate to vigorous physical activity pattern and unhealthy eating behavior pattern.

Lifestyle 2 (SAP + HEBP): A lifestyle that includes sedentary activity pattern and healthy eating behavior pattern.

Lifestyle 3 (SAP + UHEBP): A lifestyle that includes sedentary activity pattern and unhealthy eating behavior pattern.

### Statistical analysis

SPSS23.0 statistical software was used for data analysis. Quantitative variables were described as mean ± standard deviation, and the qualitative variables were described as rates or proportion (%). *t*-test was used to analyze the differences in quantitative variables between sex. Chi-square test was used to analyze the difference in qualitative variables between two groups. Pearson correlation and multiple linear regression were used to analyze association between BMI, physical activity factor, eating behavior factor and blood pressure. Logistic regression model was used to analyze the associations between overweight or obesity, physical activity patterns, eating behavior patterns and HBP in children and adolescents. *P* < 0.05 was statistically significant.

## Results

A total of 40 (5.8%) were detected with HBP, and was 5.69% (21/369) in boys and 5.97% (19/318) in girls, respectively. The SBP, MVPAF, and UHEBF in boys were higher than those in girls *(P* < 0.01), however, the SAF in girls was higher than that in boys (*P* < 0.05). The proportion of boys with MVPAF was higher than that of girls with MVPAF (*P* < 0.05). Details were shown in Table 2.

**Table 2:** Comparisons of physical activity, eating behavior, and blood pressure between sex

Variables	Girls (n = 318)	Boys (n = 369)	*t*	*P*
SBP	100.2 ± 9.2	105.0 ± 23.1	3.62	0.000
DBP	65.7 ± 8.2	65.7 ± 8.9	0.13	0.898
BMI	19.8 ± 5.1	19.5 ± 3.7	-0.90	0.366
MVPAF	-0.14 ± 0.81	0.12 ± 1.13	3.45	0.001
SAF	0.10 ± 1.01	-0.08 ± 0.99	2.38	0.017
UHEBF	-0.15 ± 0.89	0.13 ± 1.06	3.80	0.000
HEBF	0.34 ± 1.01	-0.04 ± 0.99	0.97	0.333
High SBP			0.20	0.654
No	315(99.1)	363(98.4)		
Yes	3(0.9)	6(1.6)		
High DBP			1.32	0.250
No	299(94.0)	354(95.9)		
Yes	19(6.0)	15(4.1)		
HBP			0.03	0.874
No	299(94.0)	348(94.3)		
Yes	19(6.0)	21(5.7)		
Physical activity patterns			17.42	0.000
MVPAP	138(43.4)	219(59.3)		
SAP	180(56.6)	150(40.7)		
Dietary patterns			1.30	0.254
HEBP	175(55.0)	219(59.3)		
UEBP	143(45.0)	150(42.7)		

SBP, systolic blood pressure; DBP, diastolic blood pressure; BMI, body mass index; MVPAF, moderate to vigorous physical activity factor; SAF, sedentary activity factor; UHEBF, unhealthy eating behavior factor; HEBF, healthy eating behavior factor; HBP, high blood pressure; MVPAP, moderate to vigorous physical activity pattern; SAP, sedentary activity pattern; HEBP, healthy eating behavior pattern; UEBP, unhealthy eating behavior pattern

In girls, there were positive associations between SAF, BMI and SBP, between BMI and DBP, between MVPAF and HEBF (*P* < 0.05). In boys, the MVPAF was negatively correlated with DBP (*P* < 0.05), and the SAF was positively correlated with SBP, DBP, HEBF, respectively (*P* < 0.05), and was negatively correlated with UHEBF (*P* < 0.05). Details were shown in Table 3.

**Table 3:** Coefficients of correlation among SBP, DBP, physical activity factors, eating behavior factors, BMI using Pearson correlation

Variables	SBP	DBP	MVPAF	SAF	UHEBF	HEBF
Girls						
DBP	0.599^**^					
MVPAF	-0.017	-0.104				
SAF	0.139^*^	0.104	-0.018			
UHEBF	-0.038	0.030	-0.102	-0.080		
HEBF	0.037	-0.021	0.182^**^	-0.003	0.000	
BMI	0.208^**^	0.145^**^	-0.048	-0.055	-0.023	-0.066
Boys						
DBP	0.550^**^					
MVPAF	0.013	-0.114^*^				
SAF	0.207^**^	0.152^**^	0.030			
UHEBF	-0.017	-0.024	-0.090	-0.135^**^		
HEBF	0.050	0.039	0.100	0.253^**^	0.001	
BMI	0.253^**^	0.321^**^	-0.014	0.025	-0.019	0.052

^*^P < 0.05; ^**^P < 0.01. SBP, systolic blood pressure; DBP, diastolic blood pressure; MVPAF, moderate to vigorous physical activity factor; SAF, sedentary activity factor; UHEBF, unhealthy eating behavior factor; HEBF, healthy eating behavior factor; BMI, body mass index

After adjusting for the BMI, SBP and DBP as dependent variables, MVPAF, SAF, HEBF, UHEBF as independent variables, multiple linear regression models were conducted. In girls, the SAF was positively correlated with SBP (*β*(*SE*) = 0.14 (0.50), *P* = 0.016), and the BMI was positively correlated with SBP and DBP, respectively (*β*(*SE*) = 0.22 (0.10), *P* = 0.000; *β*(*SE*) = 0.15 (0.09), *P* = 0.008). In boys, the SAF was positively correlated with SBP and DBP, respectively (*β*(*SE*) = 0.21 (1.22), *P* = 0.000; *β*(*SE*) = 0.14 (0.49), *P* = 0.006); the MVPAF was negatively correlated with DBP (*β*(*SE*)=-0.11 (0.40), *P* = 0.022); the BMI was positively correlated with SBP and DBP, respectively (*β*(*SE*) = 0.25 (0.31), *P* = 0.000; *β*(*SE*) = 0.32 (0.12), *P* = 0.000). There were no significant correlations between eating behavior factors and SBP, DBP. See Table 4 for details.

**Table 4:** Associations between physical activity factors, eating behavior factors, BMI and SBP, DBP using multiple linear regression

Dependent variables	Independent variables	β	SE	t	p	95%CI	
SBP in boys	MVPAF	0.01	1.03	0.25	0.803	-1.76	2.27
	SAF	0.21	1.22	3.95	0.000	2.42	7.20
	UHEBF	0.02	1.18	0.29	0.770	-1.98	2.67
	HEBF	-0.01	1.20	-0.28	0.783	-2.69	2.03
	BMI	0.25	0.31	4.96	0.000	0.94	2.16
DBP in boys	MVPAF	-0.11	0.40	-2.29	0.022	-1.67	-0.13
	SAF	0.14	0.49	2.76	0.006	0.39	2.33
	UHEBF	-0.01	0.45	-0.20	0.843	-0.98	0.80
	HEBF	0.01	0.46	0.002	0.999	-0.89	0.90
	BMI	0.32	0.12	6.41	0.000	0.53	0.99
SBP in girls	MVPAF	-0.02	0.64	-0.29	0.770	-1.44	1.07
	SAF	0.14	0.50	2.42	0.016	0.37	2.34
	UHEBF	-0.02	0.51	-0.41	0.681	-1.21	0.79
	HEBF	0.06	0.51	0.99	0.324	-0.50	1.51
	BMI	0.22	0.10	3.99	0.000	0.20	0.59
DBP in girls	MVPAF	-0.10	0.57	-1.64	0.103	-2.07	0.19
	SAF	0.11	0.45	1.86	0.063	-0.05	1.74
	UHEBF	0.03	0.46	0.58	0.562	-0.63	1.16
	HEBF	0.01	0.46	0.10	0.919	-0.86	0.96
	BMI	0.15	0.09	2.66	0.008	0.06	0.41

SBP, systolic blood pressure; DBP, diastolic blood pressure; MVPAF, moderate to vigorous physical activity factor; SAF, sedentary activity factor; UHEBF, unhealthy eating behavior factor; HEBF, healthy eating behavior factor; BMI, body mass index

The prevalence of high DBP and HBP in girls with overweight or obesity were significantly higher than those in girls without overweight or obesity (*P* < 0.05). In addition, the prevalence of high DBP and HBP in boys with SAP were significantly higher than those in boys with MVPAP (*P* < 0.05). Among boys and girls, eating behavior patterns and lifestyles (physical activity patterns + eating behavior patterns) were not statistically associated with high SBP, high DBP, and HBP (*P* < 0.05). See Table 5 for details.

**Table 5:** Overweight or obesity, physical activity patterns, eating behavior patterns associated with high SBP, high DBP, HBP.

Group	Girls				Boys			
	N	High SBP(%)	High DBP(%)	HBP(%)	N	High SBP(%)	High DBP(%)	HBP(%)
Physical activity patterns								
MVPAP	138	0.7	4.3	4.3	219	0.9	2.3	3.2
SAP	180	1.1	7.2	7.2	150	2.7	6.7	9.3
*χ* ^*2*^		0.13	1.15	1.15		1.71	4.39	6.25
*P*		0.724	0.284	0.284		0.191	0.036	0.012
Eating behavior patterns								
HEBP	175	5.7	5.1	5.1	219	1.4	5.0	6.4
UHEBP	143	1.4	7.0	7.0	150	2.0	2.7	4.7
*χ* ^*2*^		0.58	0.480	0.480		0.22	1.27	0.49
*P*		0.448	0.489	0.489		0.638	0.260	0.482
Lifestyles								
MVPAP + HEBP	64	1.5	4.7	4.7	99	1.0	2.0	3.0
MVPAP + UHEBP	74	1.3	4.1	4.1	120	0.8	2.5	3.3
SAP + HEBP	79	1.2	8.9	8.9	51	3.9	3.9	7.8
SAP + UHEBP	101	0.1	5.9	5.9	99	2.0	8.1	10.1
*χ* ^*2*^		1.06	1.85	1.85		2.48	5.91	6.58
*P*		0.787	0.605	0.605		0.478	0.116	0.087
Overweight or obesity								
Yes	92	2.2	10.8	10.8	129	3.1	5.4	8.5
No	226	0.4	4.0	4.0	240	0.8	3.3	4.2
*χ* ^*2*^		2.10	5.52	5.52		2.70	0.94	2.97
*P*		0.148	0.019	0.019		0.101	0.332	0.085

SBP, systolic blood pressure; DBP, diastolic blood pressure; HBP, high blood pressure; MVPAP, moderate to vigorous physical activity pattern; SAP, sedentary activity pattern; HEBP, healthy eating behavior pattern; UHEBP, unhealthy eating behavior pattern

**Fig. 1:**
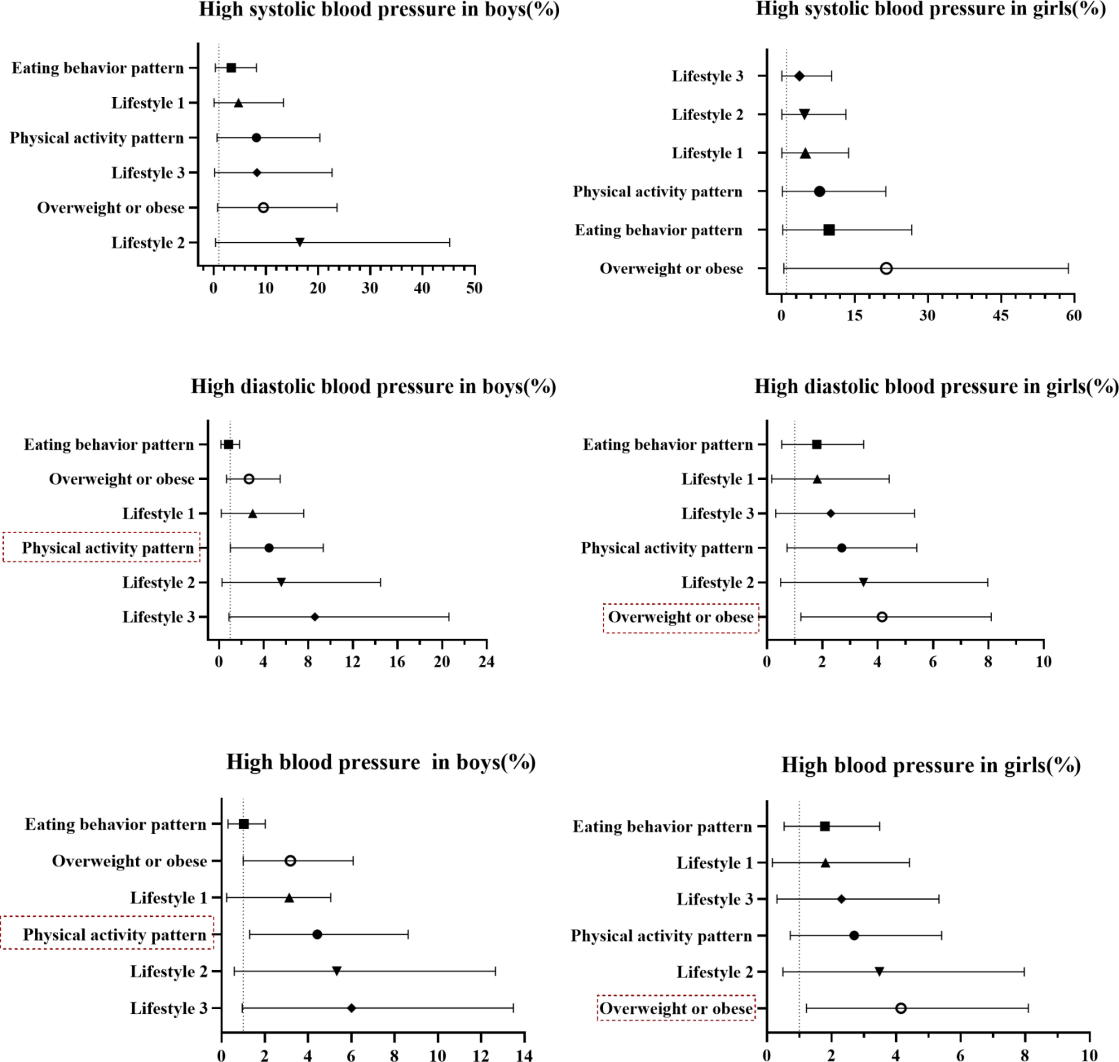
OR (95% CI) of associations between physical activity patterns, eating behavior patterns, lifestyles and high SBP, high DBP, HBP. SBP, systolic blood pressure; DBP, diastolic blood pressure; HBP, high blood pressure; lifestyle 1, moderate to vigorous physical activity pattern and unhealthy eating behavior pattern; lifestyle 2, sedentary activity pattern and healthy eating behavior pattern; lifestyle 3, sedentary activity pattern and unhealthy eating behavior pattern

After adjusting for the overweight or obesity ( overweight or obesity = 1, non-overweight or obesity = 0), high SBP (yes = 1, no = 0), high DBP (yes = 1, no = 0) and HBP (yes = 1, no = 0) as dependent variables, physical activity patterns (MHPAP = 0, SPAP = 1), eating behavior patterns (UHEBP = 1, HEBP = 0 ), and lifestyle (lifestyle 0: MHPAP + HEBP = 0, lifestyle 1: MHPAP + UHEBP = 1, lifestyle 2: SPAP + HEBP = 2, lifestyle 3: SPAP + UHEBP = 3 ) as independent variables, logistic regression models were conducted. The results showed that boys with SPAP increased the risk of HBP (OR (95% CI):3.34 (1.30–8.63)) and high DBP (OR (95% CI):3.08 (1.02–9.34)) compared with boys with MHPAP. Girls with overweight or obesity increased risk of HBP (OR (95% CI):3.14 (1.22–8.10)) and high DBP (OR (95% CI):3.14 (1.22–8.10) compared with girls without overweight or obesity. See Fig. 1 for details.

## Discussion

It has been well known that physical activities and eating behaviors are closely related to children’s blood pressure. However, children’s daily lifestyles may include both moderate to vigorous or sedentary activities, and may be accompanied by healthy or unhealthy eating behaviors, which should be a lifestyle pattern dominated by certain behaviors. Our study found that boys with sedentary activity pattern increased the risk of HBP and high DBP compared with boys with moderate to vigorous physical activity pattern.

Using EFA statistical method, we extracted MVPAF and SAF from physical activities, and extracted HEBF and UHEBF from eating behaviors. These factors can not only highlight the characteristics of physical activities or eating behaviors, but also reflect all physical activities or dietary behaviors (for example, MVPAF mainly reflects moderate to vigorous physical activities, but also includes sedentary activities), which is closer to children’s lifestyle pattern. The results of this study showed that SAF were positively associated with SBP in boys and girls, and were also positively associated with DBP in boys; SAP increased the risk of HBP and high DBP than MVPAP in boys. Some conclusions from these results could be indicated that the physical activity pattern dominated by sedentary activity will still increase levels of SBP or DBP, and even increase the risk of HBP and high DBP. The previous studies showed that sedentary activities were associated with cardiovascular system status in children [24–26]. Karatzi et al. [27] showed that sedentary activity time was associated with an increased risk of simple systolic hypertension and simple diastolic hypertension in boys. A Greek study [28] found that hypertension was associated with sedentary activity behaviors (such as reading and writing, looking at electronic screens, playing video games, etc.) in boys. The increase in SBP level may be related to the triggering of the renin - angiotensin - aldosterone system by the decrease of venous return under sedentary activity [29, 30]. In addition, reducing sedentary activity time may promote a healthier vascular status in children [31–33].

Our study did not find an association between the SAF and DBP in girls, which may be because girls’ longer sedentary activity time [34, 35] enhances their physical adaptability to reduce the response of sedentary activity to DBP. Moreover, our findings also showed that the SAF in girls was higher than that in boys, indicating that girls seem to prefer sedentary activities. We also found positive associations between BMI and SBP, DBP, and girls with overweight or obesity had increased risks of HBP and high DBP compared with girls without overweight or obesity. Obesity is an important risk factor for hypertension in children [36, 37], and also plays a role in the relationship between physical activity and blood pressure [38]. It may be because of the above reasons that there is no association between sedentary activity and DBP in girls.

The present study showed that the MVPAF was negatively associated with DBP and that the MVPAP reduced the risk of HBP relative to the SAP in boys. Most studies have shown that moderate to vigorous physical activities are protective factors for hypertension [39–41]. Grewal et al. showed that diastolic function of the left ventricle was independently associated with exercise capacity [42]. A meta-analysis on the combined effect of various exercise training on blood pressure showed that combined training reduced DBP [43]. However, the effect of exercise behavior on DBP was not reflected in girls, which may be related to differences in dynamic exercise adaptation by sex. Women appear to have less vasoconstriction and lower vascular resistance after exercise compared to men [44]. Lu et al. [45] found that the improvement effect of moderate to vigorous exercise on male patients with hypertension was better than that of female patients. Thus, the MVPAF also contributed to the healthy blood pressure in boys.

In the present study, we did not find correlations between eating behavior patterns and blood pressure in children. It has been noted that increased intake of sugar and sodium is associated with hypertension in children[46–48]. However, we found a significant positive correlation between MVPAF and HEBF in girls and a negative correlation between SAF and UHEBF and a positive correlation between SAF and HEBF in boys. Avila [49] et al. also showed that different levels of physical activity were associated with control of emotional eating. Perhaps because of the potential association between physical activity and eating behavior, there was no correlation between eating behavior pattern and blood pressure in children. In future, we need to further explore their relationship.

Several limitations of this article should be considered. Firstly, this cross-sectional study has limitations in determining the causality associations between physical activity patterns and HBP in children and adolescents, which needs to be validated by cohort or intervention studies. Secondly, genetic factors affecting blood pressure were not considered in this study, which may lead to a bias in the findings. Thirdly, blood pressure was measured only twice in this study, which could generate misclassification bias. Finally, the population involved in this study was only Chinese children and adolescents, and there are too many differences between 8-year-old children and 15-year-old adolescents (mental, nutritional, social, developmental, physiological - see, for example, natural differences in blood pressure that make it impossible to put them all to one mean value, etc.) to mix them together in one sample.

## Conclusion

The proportion of boys with moderate to vigorous physical activity pattern was higher than that of girls, on the contrary, the proportion of girls with sedentary activity pattern was higher than that of boys. Compared with the boys with moderate to vigorous physical activity pattern, boys with sedentary activity pattern may increase the risks of HBP and high DBP. Sedentary activity factor may be positively associated with SBP in both sex while moderate to vigorous physical activity factor may be negatively associated with DBP in boys.

## Data Availability

The datasets used and/or analysis during the current study are available from the corresponding author upon reasonable request.

## Abbreviations

MVPAF: Moderate to vigorous physical activity factor
SAF: Sedentary activity factor
UHEBF: Unhealthy eating behavior factor
HEBF: Healthy eating behavior factor
HBP: High blood pressure
SBP: Systolic blood pressure
DBP: Diastolic blood pressure
BMI: Body mass index
MVPAP: Moderate to vigorous physical activity pattern
SAP: Sedentary activity pattern
HEBP: Healthy eating behavior pattern
UEBP: Unhealthy eating behavior pattern
